# Involvement of endothelial CK2 in the radiation induced perivascular resistant niche (PVRN) and the induction of radioresistance for non-small cell lung cancer (NSCLC) cells

**DOI:** 10.1186/s40659-019-0231-x

**Published:** 2019-04-16

**Authors:** Qianwen Li, Yan Zong, Ke Li, Xiaohua Jie, Jiaxin Hong, Xiaoshu Zhou, Bian Wu, Zhenyu Li, Sheng Zhang, Gang Wu, Rui Meng

**Affiliations:** 1 Cancer Center, Union Hospital, Tongji Medical College, Huazhong University of Science and Technology, Wuhan, 430022 China; 2 Pharmacy Department, Union Hospital, Tongji Medical College, Huazhong University of Science and Technology, Wuhan, 430022 China

**Keywords:** Protein kinase CK2, Ionizing radiation, Non-small cell lung cancer, NF-κB, Perivascular resistance niche (PVRN)

## Abstract

**Background:**

Tumor microenvironment (TME) plays a vital role in determining the outcomes of radiotherapy. As an important component of TME, vascular endothelial cells are involved in the perivascular resistance niche (PVRN), which is formed by inflammation or cytokine production induced by ionizing radiation (IR). Protein kinase CK2 is a constitutively active serine/threonine kinase which plays a vital role in cell proliferation and inflammation. In this study, we investigated the potential role of CK2 in PVRN after IR exposure.

**Result:**

Specific CK2 inhibitors, Quinalizarin and CX-4945, were employed to effectively suppressed the kinase activity of CK2 in human umbilical vein endothelial cells (HUVECs) without affecting their viability. Results showing that conditioned medium from IR-exposed HUVECs increased cell viability of A549 and H460 cells, and the pretreatment of CK2 inhibitors slowed down such increment. The secretion of IL-8 and IL-6 in HUVECs was induced after exposure with IR, but significantly inhibited by the addition of CK2 inhibitors. Furthermore, IR exposure elevated the nuclear phosphorylated factor-κB (NF-κB) p65 expression in HUVECs, which was a master factor regulating cytokine production. But when pretreated with CK2 inhibitors, such elevation was significantly suppressed.

**Conclusion:**

This study indicated that protein kinase CK2 is involved in the key process of the IR induced perivascular resistant niche, namely cytokine production, by endothelial cells, which finally led to radioresistance of non-small cell lung cancer cells. Thus, the inhibition of CK2 may be a promising way to improve the outcomes of radiation in non-small cell lung cancer cells.

## Introduction

Lung cancer remains the leading cause of morbidity and mortality worldwide. Radiotherapy is one of the main treatment options for lung cancer of various stage and pathological type [1], which significantly increases the local control rate as well as to improve survival. However, the intrinsic radioresistance of cancer cells and recurrences of tumors in the previous radiation field still exist [2]. Therefore, it is crucial to investigate the mechanism of radioresistance and to find novel therapeutic strategies to improve patient outcomes.

Previous works have concentrated on understanding radioresistance by focusing on mechanisms within tumor cells. Although recent evidence suggests that the tumor microenvironment (TME) plays an important role in determining outcomes of radiotherapy [3–5]. TME includes tumor cells, fibroblasts and immune cells, as well as secretory products (such as cytokines and chemokines) and non-cellular components in extracellular matrix of these cells. It has been reported that the perivascular resistance niche (PVRN), which is formed by paracrine cytokine production in endothelial cells after radiation, can regulate radioresistance of tumor cells [6, 7]. However, the exact underlying molecular mechanisms of PVRN are largely unknown.

A number of studies have shown that nuclear transcription factor (NF-κB) regulates the expression of a series of cytokine genes and is considered to be the center of molecular regulation of cytokine release [8]. NF-κB is widely expressed in various vascular endothelial tissues [9], and can be activated by radiation and involved in the adaptive response of cells to radiation [10]. Radiation can increase the concentration of a variety of cytokines including IL-8 and IL-6. IL-6 was reported to be highly expressed in tumors and can promote the growth of tumors and increase tumor resistant to treatment [11–13]. IL-8 was imported to be implicated in many cell processes including angiogenesis, cell proliferation and metastasis [14].

Protein kinase CK2 is a highly conserved serine/threonine protein kinase [15]. It is able to phosphorylate more than three hundreds different substrates, including NF-κB [16]. CK2 can phosphorylate p65 subunit of NF-κB [17–19], as well as IκB-α [20], which is degraded after phosphorylation, to promote NF-κB nuclear translocation and activate its transcriptional activity [21].

In our present study, we investigated the involvement of vascular endothelial cells in radioresistance of NSCLC cells following exposure to ionizing radiation (IR) and further explored the effect of CK2 inhibition on this process.

## Materials and methods

### Cell culture and reagents

Human non-small cell lung cancer cells A549, H460 and human umbilical vein endothelial cells (HUVECs) was purchased from the Chinese Academy of Science Committee on Type Culture Collection Cell Bank (Shanghai, China). Cells were cultured in 5% CO_2_ at 37 °C. A549 and H460 cells were cultured in RPMI 1640 (Gibco, CA, USA) supplemented with 10% FBS (fetal bovine serum, Gibco, CA, USA) and 1% penicillin/streptomycin (Gibco, CA, USA). HUVECs cells were cultured in endothelial cell medium (Sciencell, USA). Quinalizarin was purchased form Merck (Germany), CX-4945 was purchased from Selleck (Houston, TX, USA).

### Ionizing radiation (IR) treatment

X-rays were generated by a linear accelerator (Primus K, Siemens, Munich, Bayern, Germany) with 6 MV photons/100 cm focus-surface distance and a dose rate of 2.0 Gy/min.

### In vitro kinase assay

HUVECs were treated with DMSO, 25 μM Quinalizairn or 10 μM CX-4945 for 1 h, 6 h or 24 h, lysed and extracts were used in a kinase filter assay. The kinase assay was performed as described previously [22].

### Western blot

Nuclear and cytosolic proteins of HUVECs were extracted using the nuclear and cytoplasmic protein extraction kit (Beyotime, Shanghai, China) or the total proteins were extracted, separated on SDS gel and then transferred onto PVDF membranes. After blocked with 5% nonfat dry milk, the blots were incubated with anti-CK2 α, α′ and β (generated as described previously [23] and was a gift from Prof. Dr. Mathias Montenarh, Universität des Saarlandes, Germany), anti-p65, anti-p-p65, anti-histone H3 (Santa Cruz Biotechnology, CA, USA) and anti-GAPDH (Abcam, Cambridge, UK) antibodies at 4 °C overnight. The blots were washed and incubated with secondary antibody (Boster, Wuhan, China). After addition of chemiluminescent reagent, the blots were exposed to imaging system (UVP ChemiDoc-It, USA). The intensities of the bands were detected by Image J.

### CCK8 assay

HUVECs were pretreated with complete medium, DMSO, 25 μM Quinalizarin or 10 μM CX-4945 for 6 h, then exposed to 4 Gy IR. The medium was replaced with fresh one before IR. After 24 h, the conditioned medium was collected and filtered, then applied to 4 Gy irradiated A549 and H460 cells in 96-well plants. Cell viability was measured by cell counting kit-8 (CCK8) assay after 72 h or 96 h. After indicated treatment cells were incubated with CCK8 solution (Boster, Wuhan, China) for 1 h at 37 °C, then absorbance was detected at 450 nm by a multimode plate reader (EnSpire, PerkinElmer, USA).

### Immunofluorescence microscopy

HUVECs were seeded onto cover slips, then pretreated with complete medium, DMSO, 25 μM Quinalizairn or 10 μM CX-4945 then irradiated with a dose of 4 Gy. After 6 h or 24 h, cells were fixed with 4% formalin, permeabilized with 5‰ Triton-X for 20 min on ice, blocked with 5% BSA for 30 min and stained with anti-p65, p-p65 (Santa Cruz Biotechnology, CA, USA) or anti-CK2α antibody at 4 °C overnight. After washing by PBS, cells were incubated with Cy3 or FITC-conjugated secondary antibody (Boster, Wuhan, China) for 1 h avoid light. Cell nuclei were stained with DAPI for 10 min. Photographs were taken by a confocal microscope (Olympus, Tokyo, Japan).

### Cytometric bead array

HUVECs were pretreated with complete medium, DMSO, 25 μM Quinalizairn or 10 μM CX-4945 then irradiated with a dose of 4 Gy. The medium was replaced with fresh one before IR and the supernatants were collected 24 h after radiation. IL-8, IL-1β, IL-6, IL-10, TNF-α, IL-12p70 concentrations were measured by the cytometric bead array (CBA) kit (BD Biosciences). 10 μl of each capture beads were added into 50 μl of each sample then mixed with 50 μl PE detection reagent. After 3 h incubation at room temperature avoid light, samples were then washed once with wash buffer, resuspended in 300 μl wash buffer and tested on a flow cytometer FACScan (BD Biosciences). Finally, data were analyzed by CBA software (BD Biosciences).

### Statistical analysis

Data were shown as mean ± SD of at lest three individual experiments. Student’s *t* test or one-way ANOVA followed by Tukey’s test was used for statistical analyses, and p < 0.05 was considered statistically significant.

## Results

### Effect of Quinalizarin and CX-4945 on CK2 kinase activity and cell viability in HUVECs

**Fig. 1:**
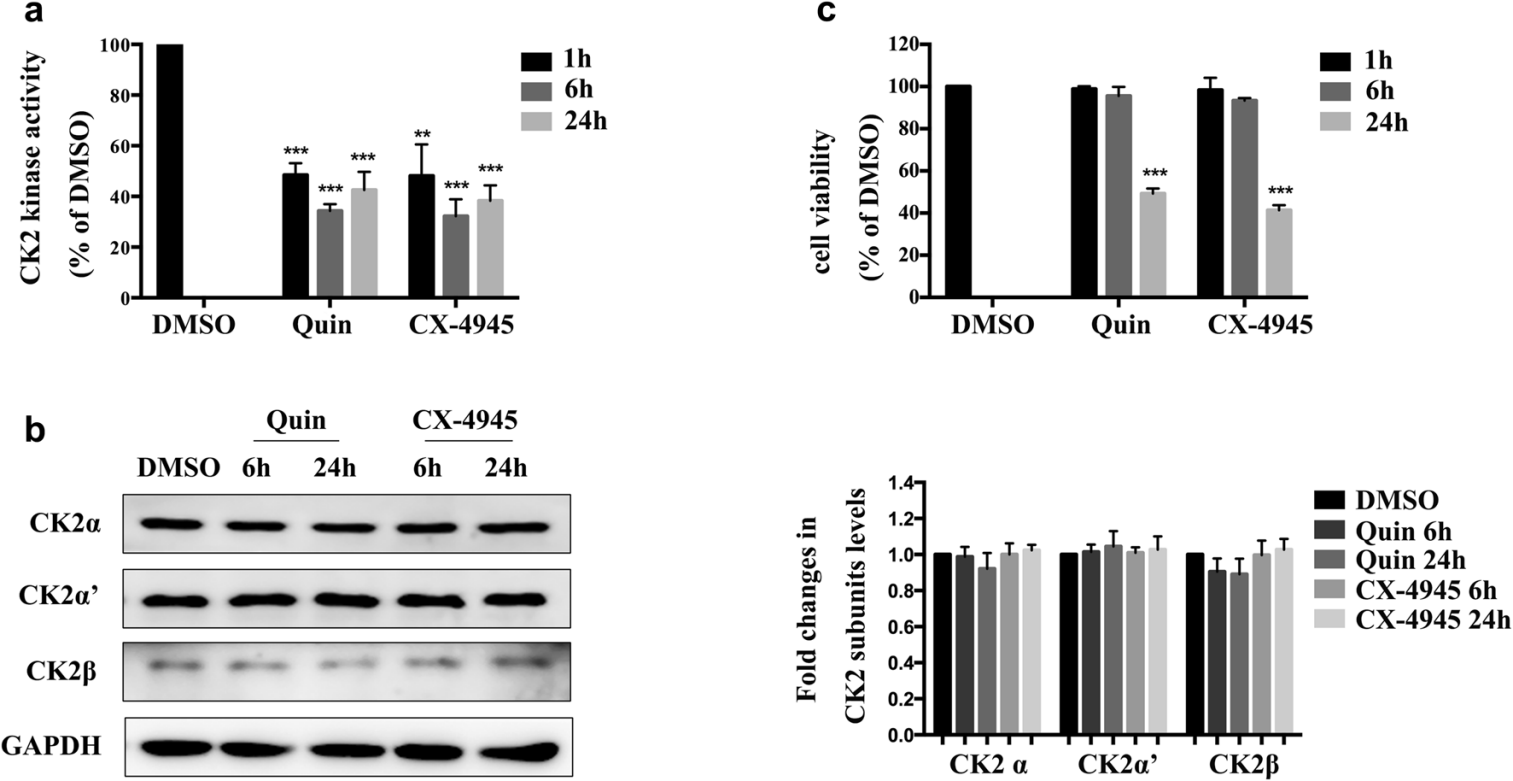
The effect of Quinalizarin and CX-4945 on CK2 kinase activity and cell viability in human endothelial cells. **a** HUVECs were treated with DMSO, 25 μM Quinalizarin, or 10 μM CX-4945 for 1 h, 6 h, or 24 h. After cell lysis, in vitro kinase assay was conducted to measure CK2 kinase activity. Mean ± SD were calculated for three independent experiments (**p < 0.01, ***p < 0.001). **b** Protein expressions of CK2 α, α′ and β subunits in HUVECs were assessed by Western blot. **c** HUVECs were treated as described above. Cell viability was determined by CCK8 assay. Mean ± SD were calculated for three independent experiments (***p < 0.001)

In the first step of the experiment, we applied two specific CK2 inhibitors, Quinalizarin and CX-4945 [24–26]. HUVECs were exposed to 25 μl Quinalizairn or 10 μl CX-4945 for 1 h, 6 h or 24 h. The results proved that both inhibitors decreased the activity of CK2 by about 50% or more at all these three time points (Fig. 1a, **p < 0.01, ***p < 0.001). And both Quinalizarin and CX-4945 did not affect the protein expression of CK2 α, α′ and β subunits (Fig. 1b). CCK8 assay was conducted in order to determine the cell viability after CK2 inhibition. As shown in Fig. 1c, Quinalizarin and CX-4945 did not affect the viability of HUVECs at 1 h and 6 h, but at 24 h both of two CK2 inhibitors significantly reduced the cell viability (***p < 0.001). Therefore, in the following experiments we chose 6 h as the best time point when pretreated the HUVECs with CK2 inhibitors, since it markedly suppressed the activity of CK2 without significantly affect the viability of cells.

### Inhibition of CK2 in HUVECs reverses its ionizing radiation (IR)-induced viability-promoting capacity to non-small cell lung cancer (NSCLC) cells after IR

**Fig. 2:**
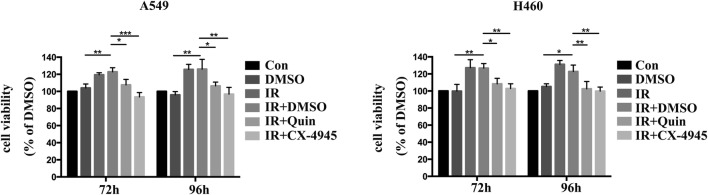
Inhibition of CK2 in HUVECs reverses its ionizing radiation (IR)-induced viability-promoting capacity to non-small cell lung cancer (NSCLC) cells after IR. HUVECs were pretreated with complete medium, DMSO, 25 μM Quinalizarin or 10 μM CX-4945 for 6 h, then exposed to 4 Gy IR. The medium was replaced with fresh one before IR. After 24 h, the conditioned medium was collected and filtered, then applied to 4 Gy irradiated A549 and H460 cells, and continuously cultured for 72 h or 96 h, then cell viability was measured by CCK8 assay. Mean ± SD were calculated for three independent experiments (*p < 0.05, **p < 0.01, ***p < 0.001)

As long been considered that tumor microenvironment (TME) affects the radiosensitivity of tumor cells and the endothelial cells are important components of the TME. We first investigated the role of endothelial cells on the resistance niche of NSCLC cells after exposure to IR. HUVECs were applied and were pretreated with complete medium, DMSO, Quinalizarin or CX-4945 for 6 h and then exposed to IR, finally the supernatant from HUVECs was collected, filtered and applied to irradiated A549 or H460 cells. As shown in Fig. 2, incubation with the supernatant from the IR-exposed HUVECs for 72 h or 96 h enhanced the cell viability of irradiated A549 and H460 cells as compared with the DMSO group (p = 0.0022, p = 0.0013, for A549; p = 0.0028, p = 0.0203, for H460). However, pretreatment of HUVECs with both Quinalizarin or CX-4945 obviously slowed down such cell viability increment at 72 h (p = 0.0115, p < 0.0001, for A549; p = 0.0432, p = 0.0074, for H460) and 96 h (p = 0.0315, p = 0.0017, for A549; p = 0.0077, p = 0.0030, for H460). Collectively, these results indicated that endothelial cells, such as HUVECs, when exposed to IR could secrete and form a microenvironment or niche to promote the cell viability of NSCLC cells. Specific CK2 inhibitors could reverse such promotion environment and finally reduced the growth enhancement of NSCLC cells.

### Inhibition of CK2 decreased IR induced IL-8 and IL-6 secretion in HUVECs

**Fig. 3:**
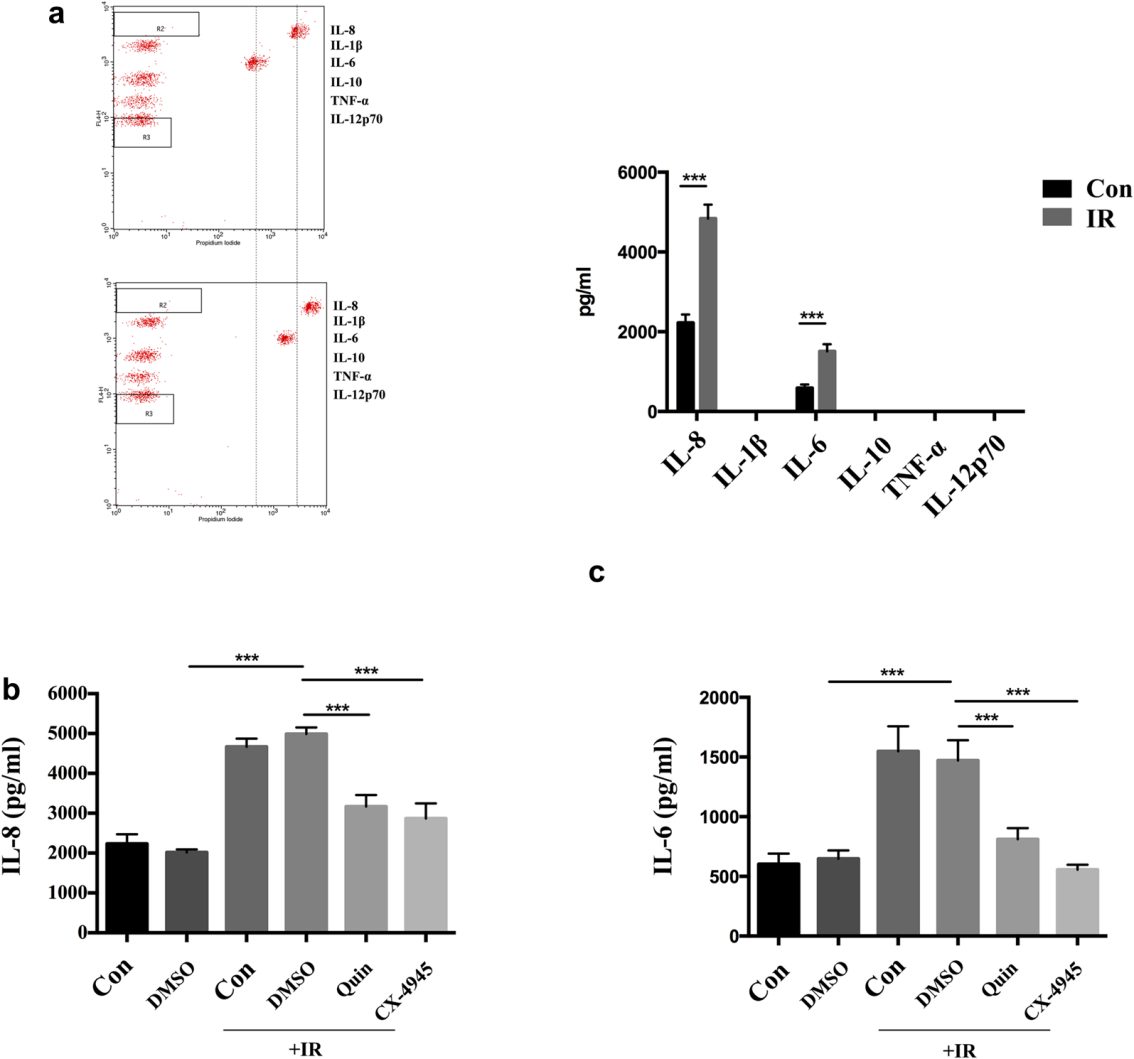
CK2 inhibition decreased IR induced IL-8 and IL-6 secretion. **a** HUVECs was exposed to 4 Gy IR. Conditioned medium was collected 24 h after radiation. Then the concentration of IL-8, IL-1β, IL-6, IL-10, TNF-α, IL-12p70 were detected using a CBA kit. Mean ± SD were calculated for three independent experiments (***p < 0.001). HUVECs were pretreated with complete medium, DMSO, 25 μM Quinalizarin or 10 μM CX-4945 for 6 h, then exposed to 4 Gy IR. Conditioned medium was collected 24 h after radiation. Concentration of **b** IL-8 and **c** IL-6 were detected using a CBA kit. Mean ± SD were calculated for three independent experiments (***p < 0.001)

To further elucidate the exact cytokine produced by HUVECs to form the above mentioned resistant niche, we, in the next step, measured the expression of IL-8, IL-1β, IL-6, IL-10, TNF-α, IL-12p70 in the supernatant of HUVECs after IR exposure. As shown in Fig. 3a, after exposed to IR the expression of IL-8 and IL-6 was significantly elevated (p < 0.0001, p < 0.0001). In order to check whether CK2 was involved in the release process, the specific CK2 inhibitor was applied. Results showing that either Quinalizarin or CX-4945, could markedly decrease the increment of IL-8 and IL-6 production induced by IR (p < 0.0001, p < 0.0001, for IL-8; p = 0.0004, p < 0.0001, for IL-6), with CX-4945 had the greatest release inhibition effect (Fig. 3b, c). Collectively, these results suggested that IR exposure induced IL-8 and IL-6 production in endothelial cells which could be markedly affected by CK2 inhibition, indicating a possible role of CK2 in cytokine production after IR exposure.

### Inhibition of CK2 inhibition decreased IR induced phosphorylated p65 expression in HUVECs

**Fig. 4:**
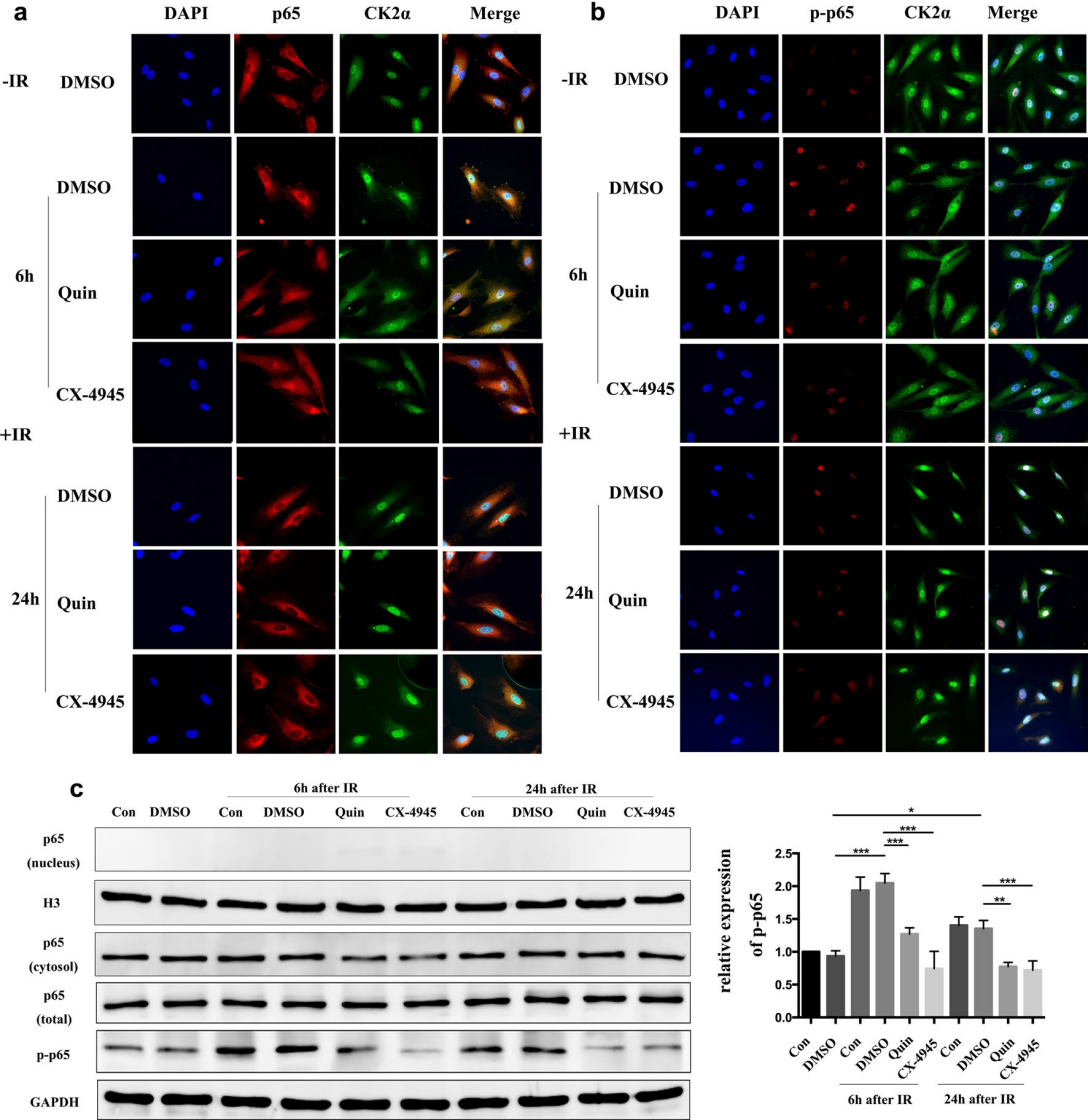
CK2 inhibition decreased IR induced phosphorylated p65 expression in HUVECs. HUVECs were pretreated with complete medium, DMSO, 25 μM Quinalizarin or 10 μM CX-4945 for 6 h, then exposed to 4 Gy IR. Cells were fixed 6 h or 24 h after IR and incubated with primary antibodies. **a** p65 and **b** p-p65 was labeled by Cy3-conjugated secondary antibody and CK2 α was labeled by FITC-conjugated secondary antibody. Cell nuclei were stained with DAPI. Fluorescence images were taken at ×400 magnification by a scanning confocal microscope. **c** Protein expressions of nuclear and cytoplasmic p65, total p65 and p-p65 in HUVECs, which were treated as described above, were assessed by Western blot. Mean ± SD were calculated for three independent experiments (*p < 0.05, **p < 0.01, ***p < 0.001)

To further explore the exact role of CK2 in endothelial cells on forming the resistant niche of NSCLC cells after IR, we detected the relationship between CK2 and NF-κB, which was considered as the master molecule in cytokine production. The subcellular localization, namely shuttling from cytoplasm to nucleus, of p65 or the expression of phosphorylated p65, as the indication of NF-κB activation, in endothelial cells was detected after exposed to IR and/or CK2 inhibition. HUVECs were pretreated with CK2 inhibitors for 6 h, then exposed to IR. Cells were fixed and immunofluorecence assays were conducted thereafter. As illustrated in Fig. 4a, p65 localized solely in the cytoplasm in untreated HUVECs. IR exposure did not affect the localization of p65 in HUVECs and the result was consistent with the Western bolt assay (Fig. 4c). However, the combination of IR and CK2 inhibitors seems to slightly increase the expression of p65 in the nucleus at 6 h after IR (Fig. 4a). In addition, the CK2 catalytic subunit CK2α was also detected. It was shown that CK2α expressed mainly in the nucleus and its subcellular localization was not affected by IR or inhibition of CK2.

The subcellular localization of phosphorylated p65 was also detected in HUVECs by the phospho-specific antibodies against the serine 529 which was regarded as a CK2 phosphorylation site [18, 27]. As shown in Fig. 4b, phosphorylated p65 was mainly expressed in the nucleus. IR exposure obviously increased the nuclear signal of phosphorylated p65, while CK2 inhibition markedly reduced its expression. We also conducted western bolt to measure the expression of p65 and phosphorylated p65, as shown in Fig. 4c, the expression levels of p-p65 were significantly increased 6 h or 24 h after exposed to IR (p < 0.0001, p = 0.0455), and the treatment of both Quinalizarin and CX-4945 reduced this enhancement (p < 0.0001, p < 0.0001, for 6 h; p = 0.0024, p = 0.0009, for 24 h).

**Fig. 5:**
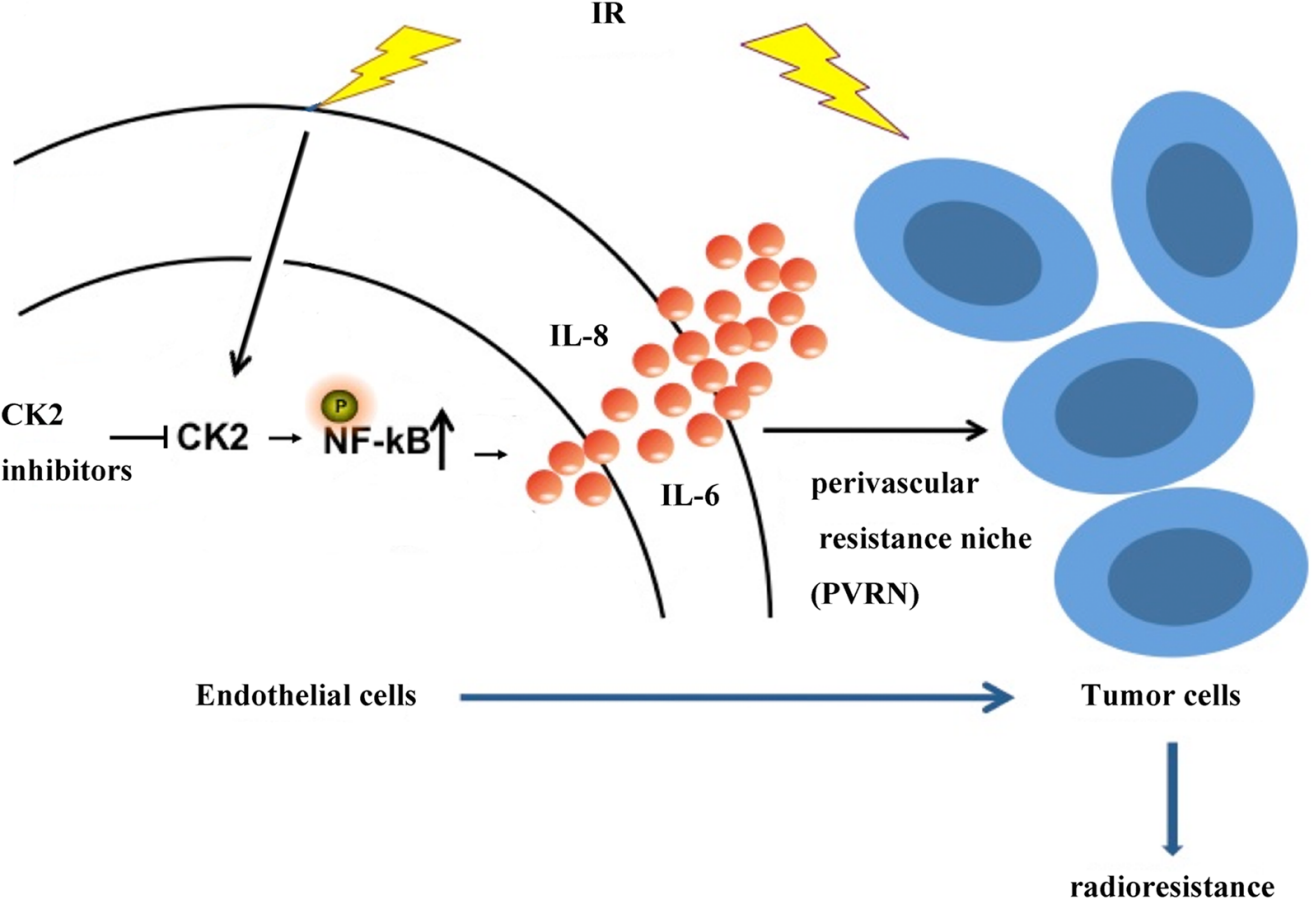
Schematic representation of a putative mechanism underlying the forming of perivascular resistance niche (PVRN) after exposure to IR. CK2 inhibition suppresses the phosphorylation of NF-κB, then reduces the increase of IL-8 and IL-6 in endothelial cells after exposure to IR, which destroyed the forming of PVRN and finally leads to radiosensitize lung cancer cells (→ indicates stimulation effect, ┫means inhibitory effect)

In summary, as illustrated in Fig. 5, CK2 inhibition was able to suppress the phosphorylation of p65, then reduced the increase of IL-8 and IL-6 in endothelial cells after exposure to IR, which destroyed the forming of the perivascular resistance niche (PVRN) and finally led to radiosensitize lung cancer cells.

## Discussion

Ionizing radiation (IR) induced tumor microenvironment remodeling has a great impact on the outcomes of radiotherapy in cancer. The mechanisms underlying such process include direct contact with tumor cells and their surrounding stroma, the interaction of tumor cells and host immune cells, vascular endothelial cells or fibroblasts, and the paracrine effects of cytokines’ secretion [3].

Protein kinase CK2 is reported to be highly expressed in lung cancer cells and tissues [28, 29]. The inhibition of CK2 in lung cancer cells effectively suppresses the proliferation of tumor cells [24, 30–33]. Down regulation of CK2 is also reported to enhance the radiosensitivity of nasopharyngeal carcinoma cells [34]. In addition, in our previous study we verified that CK2 inhibition could radiosensitize lung cancer cells [22]. However, these researches focused on mechanisms solely within tumor cells, in this study, we explored the role of CK2 in IR induced tumor microenvironment remodeling process and further investigated the effects of endothelial CK2 inhibition on the radiosensitivity of lung cancer cells. Our results found that HUVECs, when exposed to IR could secrete and form a microenvironment or niche to promote the cell viability of NSCLC cells. Specific CK2 inhibitors, Quinalizarin and CX4945, could reverse such promotion environment and finally reduced the paracrine growth enhancement of NSCLC cells.

Regarding the exact mechanisms underlying such process, in the present study we found that after exposed to IR, secretion of cytokines such as IL-8 and IL-6 significantly elevated from endothelial cells, which could be markedly affected by CK2 inhibition (Fig. 3b, c). Similarly, as previously reported in other cell lines, IL-6 was a multifunctional cytokine which was induced by radiation [35, 36] and played pivotal role in the process of radioresistance [37]. IL-8 was also described in nasopharyngeal carcinoma cells to be a crucial player in mediating radiation response and induction of radioresistance [38]. In addition, there was also evidence showing that IL-6 expression could be modulated by CK2 in inflammatory breast cancer cells [39]. IL-8 and IL-6 was reported to increase the cell proliferation of NSCLC cells. It indicated that the increased cell viability of NSCLC cells co-cultured with irradiated HUVECs supernatant (Fig. 2) was associated with the enhanced secretion of IL-8 and IL-6 after irradiation. Our results were to date the first to show, in endothelial cells, the irradiation induced expression of cytokines such as IL-8 and IL-6 was controlled or mediated by protein kinase CK2.

A growing body of literature proves that NF-κB activation can increase the secretion of major inflammatory factors, including IL-6, IL-8, TNF-α, IL-1, etc. Such inflammatory factors are also act as potent activators for NF-κB. This positive feedback loop between NF-κB and inflammation improve cell proliferation and transformation [40, 41]. The transcription factor p65 is a component of the heterodimeric NF-κB complex. Phosphorylation of p65 activates its transcriptional activity. Protein kinase CK2 is an important regulator of p65, which can phosphorylate p65 at Ser 529 to increase the transcriptional activity of it [18, 27]. CK2 can also phosphorylate IκBα to promote the degradation of IκBα and activate NF-κB [20]. In the current study, we detected an increase of phosphorylated p65 in HUVECs at 6 h and 24 h after IR. And the pretreatment of either CX-4945 or Quinalizarin suppressed it (Fig. 4b, c). In addition, we also found that CK2 inhibitors slightly enhanced the level of nuclear p65. This hypo-phosphorylation caused accumulation of p65 in the nucleus was mainly due to a defective IκBα synthesis [42]. This was in line with the results published by Ampofo et al. that CK2 inhibition suppresses TNF-α induced NF-κB p65 phosphorylation at Ser529 in Human Dermal Microvascular Endothelial Cells [43].

## Conclusion

CK2 inhibition can reverse the perivascular resistance niche (PVRN) after IR by reduces the IR enhanced phosphorylation of p65 and the expression of IL-8 and IL-6 in HUVECs. This might be a promising way for CK2 inhibitors to enhance the radiosensitivity of non-small cell lung cancer cells.

## Abbreviations

CK2: protein kinase CK2
PVRN: perivascular resistant niche
NSCLC: non-small cell lung cancer
TME: tumor microenvironment
IR: ionizing radiation
HUVECs: human umbilical vein endothelial cells
